# Regulation of Spontaneous Propagating Waves in the Embryonic Mouse Brainstem

**DOI:** 10.3389/fncir.2016.00110

**Published:** 2017-01-04

**Authors:** Martha M. Bosma

**Affiliations:** Department of Biology, University of WashingtonSeattle, WA, USA

**Keywords:** brainstem, embryo, mammalian, serotonin, mouse model, wave propagation

## Abstract

Spontaneous activity (SA) modulates many aspects of neural development, including neuronal phenotype, axon path-finding and synaptic connectivity. In the embryonic mouse brainstem, SA initially is recorded in isolated cells at embryonic day (E) 9.5, and 48 h later takes the form of propagating waves. The majority of these waves originate from one midline initiation zone (InZ), which is situated within the developing serotonergic raphe. InZ cells express a *t*-type calcium channel, are depolarized, and have high membrane resistance, the combination of which allows spontaneous depolarization. Propagating events require signaling at metabotropic 5-HT receptors; a possible source could be 5-HT released by newly differentiating 5-HT neurons. At E11.5, waves propagate throughout the hindbrain, with some events crossing into the midbrain. At E12.5, lateral cells (further than 150 μm from the midline) up-regulate expression of a K channel that increases resting conductance and hyperpolarizes them, preventing the propagation of waves laterally. At the same stage, cells in the isthmus up-regulate *t*-type calcium channels, permitting more events to cross into the midbrain, some of which form recurring loops of activity that are able to keep intracellular calcium levels high for many minutes. At E13.5, caudal hindbrain cells hyperpolarize utilizing the same K conductance, and 24 h later, at E14.5, the InZ hyperpolarizes and no longer undergoes spontaneous events. Thus, 5-HT receptor-dependent propagating waves in the embryonic brainstem are generated and propagated by regulation of membrane conductance. We discuss these mechanisms, and the possible role of this SA in neuronal development.

## Introduction

Spontaneous activity (SA) in the developing brain influences proliferation, specification and synaptic connectivity of neurons during formation of brain circuits (Moody and Bosma, 2005; Blankenship and Feller, 2010; Rosenberg and Spitzer, 2011). Our lab has explored the expression patterns and mechanisms of SA in the embryonic mouse brainstem during the period of neuronal specification, differentiation and axon pathfinding within that structure. This review will cover the known mechanisms regulating brainstem SA, highlighting the unique attributes of SA at each stage.

**Figure 1:**
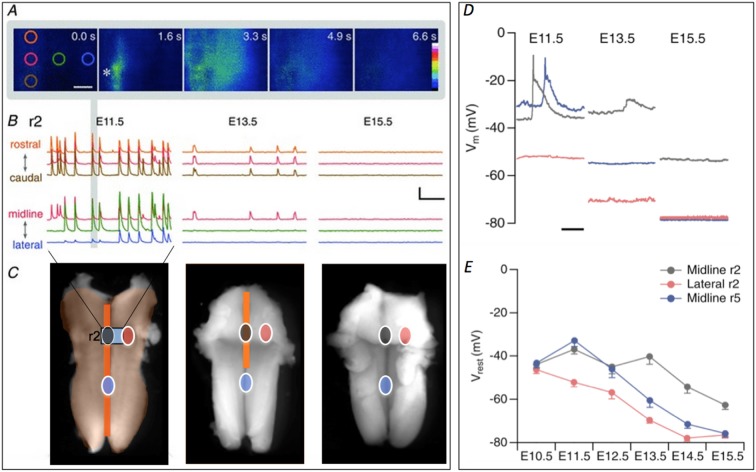
**Retraction of spontaneous activity (SA) over developmental time. (A)** Initiation and propagation of an event at E11.5. Images taken at the indicated times show an event initiating at the initiation zone (InZ; *), which propagates rostrally and laterally. Colored circles indicate regions of interest (ROIs, at 0.0 s) positioned rostrocaudally along the midline (orange, red, brown) and mediolaterally from the InZ (red, green, blue). Scale bar: 100 μm. **(B)** Representative [Ca]_i_ traces recorded at E11.5 (left), E13.5 (middle) and E15.5 (right) in r2, showing rostrocaudal (top) and mediolateral (bottom) propagation. Traces are stacked for clarity and the color corresponds to the position of ROIs shown in **(A)**. The event from the images in **(A)** is marked with gray vertical bar. Scale bar = 5 *F*/*F*, 1 min. **(C)** At E11.5, SA encompasses the entire hindbrain. Intensity of [Ca]_i_ signal is higher at the midline (dark orange) compared with the lateral regions (light orange). The region of [Ca]_i_ imaging shown in **(A,B)** is indicated by gray rectangle, marked “r2”. By E13.5 the propagation has retracted from lateral and caudal regions middle image. SA disappears by E15.5 (right image). Circles indicating sites of patch clamp recordings shown in **(D,E)**, with gray indicating InZ (r2) site, blue is caudal midline site, and red is lateral site. Some parts of the figure are adapted and modified from Hunt et al. (2006a) and Watari et al. (2013). Scale bar: 1 mm. **(D)** Examples of current clamp recordings at different sites and stages, showing SA events in midline (black, blue) and gradual hyperpolarization over time. **(E)** Averaged data from all stages, showing hyperpolarization first in lateral (red) regions, followed by caudal midline (blue) and finally by r2 (InZ) midline cells.

**Figure 2:**
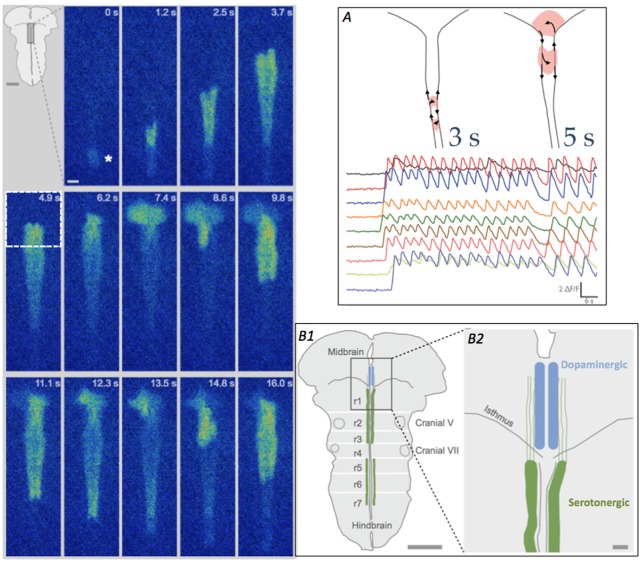
**Looping pattern of SA at E12.5. Left** Looping pattern near the isthmus shows event initiating at the InZ (*), propagating towards the isthmus and looping counterclockwise in the isthmus. After exiting the midbrain (at 7.4 s), the wave splits, with one branch re-activating the same isthmic trigger point (9.8 s) and the other branch propagating caudally along the midline. At 11.1–13.5 s, the rostral branch loops again in the midbrain. Scale bar = 100 μm. Inset. Diagram of brainstem, showing rhombomere (r) boundaries and location of 5-HT neurons in the hindbrain. **(A)** Looping can take two patterns, either within the hindbrain (left diagram, and more rapid [Ca]_i_ oscillations) or crossing into the midbrain tegmentum (right diagram and slower [Ca]_i_ oscillations); looping into the midbrain causes repetitive calcium influx in the region of differentiating dopaminergic cells. Traces show fluorescence from different recording regions along the midline of the brainstem. Time scale = 6 s. **(B1)** Diagram of brainstem, detailing region in box at 4.9 s timepoint, showing the location of the developing raphe serotonergic (green, hindbrain) and tegmental dopaminergic (blue, midbrain) neurons at E12.5. White lines indicate the locations of the former rhombomeres (r1–r7) in the hindbrain. Scale bar = 1 mm. **(B2)** Detail of the midline and isthmus shows the pathways of serotonin axons (green), putative carriers of [Ca]_i_ events on two parallel tracks flanking the midline (Rockhill et al., 2009), into the midbrain. Scale bar = 100 μm.

The brainstem comprises neurons involved in sensory input, motor control (cranial nerves), respiratory control, modulatory neurotransmitter control (dopamine, serotonin, histamine and norepinephrine), as well as axon tracts connecting the spinal cord to the rostral CNS. The pons and medulla derive from the rhombencephalon, or hindbrain, which initially is segregated into rhombomeres (r), units of development that assign lineage identity early in development. Upon dissolution of rhombomere (r) borders at embryonic day (E) 11.5, propagating waves of SA are recorded in the brainstem (Hunt et al., 2005). Most SA events (>80%) originate at the midline of the hindbrain near the r2/3 boundary, at a position that we term the initiation zone (InZ; * in Figures 1, 2). Brainstem SA is dependent on signaling through metabotropic 5-HT receptors (5-HT_2A/C_). Propagating SA is expressed between E11.5-E14.5, with the majority of waves initiated from the InZ at all stages. Midline-derived SA does not change when the spinal cord remains attached during recording, and although occurring at similar times to pre-respiratory correlated network oscillations (Abadie et al., 2000), is distinct both in timing and in mechanism of initiation.

## Developmental Timing of Brainstem SA

At E9.5–10.5, asynchronous [Ca]_i_ events are recorded in isolated cells (Gust et al., 2003). Propagating waves abruptly commence at E11.5, in which the events initiate largely at the InZ and travel throughout the brainstem, without propagating to the spinal cord. This is followed by gradual retraction of the waves over developmental time, first from lateral, then caudal regions, before ceasing completely at E14.5 (Bosma, 2010). As midbrain SA is dependent on hindbrain SA (see below), it also ceases to express propagating waves. This review will focus on the details of brainstem SA initiation, propagation and retraction, with comparisons to other mammalian brain regions in which SA is expressed.

## Identity of InZ Neurons

At E11.5, over 80% of events initiate in the InZ; waves then propagate to encompass the entire hindbrain (Hunt et al., 2005). During the window of brainstem SA, the InZ continues to initiate most events even as waves become progressively restricted from lateral, and then caudal tissue, until lastly the InZ not longer is spontaneously active. Because hindbrain SA is focused in regions where the serotonergic (5-HT) raphe is developing, we have examined the expression of those neurons during the period of SA. 5-HT neurons are specified and differentiate at the midline of the hindbrain in discrete groups: the rostral raphe (B4–9) originates in r1–3, while the caudal groups (B1–3) originate in r5–7; no serotonergic neurons are found in r4 (Wallace and Lauder, 1983; Hendricks et al., 1999; Kiyasova and Gaspar, 2011). 5-HT neurons of r1, within the rostral raphe, are specified beginning at E9.5, and begin differentiation at E10.5; those in r2 and r3 are specified and differentiate 24 h later. Caudal 5-HT neurons are also specified at E10.5, but don’t begin to differentiate until E12.5 (Deneris and Wyler, 2012). Thus, at the point in time when 5-HT-receptor dependent propagating waves are present, 5-HT cells are differentiating and expressing transmitter. At E11.5, 80% of neurons within the former r1–3 and within 125 μm of the midline (containing the InZ) are immunopositive for serotonin (Hunt et al., 2005). As 5-HT receptors are the only transmitter system regulating brainstem SA (Hunt et al., 2006a), it is likely that 5-HT released from neurons in the InZ mediate SA.

## Mechanisms of Event Initiation

InZ cells undergo spontaneous depolarizing events that are graded in amplitude; using dual patch clamp, we demonstrated that these events propagate laterally (Moruzzi et al., 2009). Midline cells initiate events due largely to the expression of intrinsic membrane conductances that permit spontaneous depolarization. Central to these is the *t*-type calcium channel, combined with relatively high resting resistance (Moruzzi et al., 2009). Transient, or *t*-type, Ca channels are voltage-activated and inactivate during a sustained voltage pulse (Perez-Reyes, 2006). Because of overlap of the activation and inactivation curves, there is a window of voltages at the depolarized end of the inactivation range at which channels have a significant probability of opening; this is called window current. Based on pharmacology and immunohistochemistry, we showed that the *t*-type Ca channel Ca_v_3.3 is expressed in the InZ, and highly expressed in commissural axons crossing the midline ventral to the floor plate. The window current of hindbrain *t*-type Ca channel peaks at approximately −31 mV (Watari et al., 2014), allowing individual Ca channels to have a significant probability of being open at the resting potential of midline cells (approximately −37 mV at E11.5; Watari et al., 2013). As the membrane resistance of these cells is high and they are sparsely electrically coupled (Moruzzi et al., 2009), depolarization generated by Ca channel opening likely results in the initiation of spontaneous events leading to propagating waves.

Events in the hindbrain are graded in amplitude, and vary across the tissue: the most rapid component recorded in midline cells is longer than 50 ms, while the plateaus range in duration from 700 ms (medially) to 1540 ms (laterally). These long-duration events are similar to depolarizing events in the earliest stage of retinal wave expression, in which starburst amacrine cells, acting through a combination of nAChR and gap junctional connections, undergo spontaneous depolarizations 1–2 s in duration (Zheng et al., 2004). Similar long-duration events are recorded in E12.5 spinal cord motoneurons, mediated by nicotinic presynaptic facilitation of GABAergic and glutamatergic depolarizing potentials (Czarnecki et al., 2014). SA includes excitation via glycinergic receptors (Hanson and Landmesser, 2003), from glycine released from radial glial progenitors (Scain et al., 2010). Unlike SA in other brain regions (retina, cortex, spinal cord), there is little synaptic input augmenting initiation of events. In whole cell recording, fluctuations indicating synaptic input are not observed at any voltage. Thus, the initiation of events is likely caused exclusively by intrinsic membrane conductance, with possible modulation via second messenger activation by 5-HT receptors.

## Transmitter Dependence

SA in the hindbrain is dependent on signaling by metabotropic 5-HT receptors; this is the only CNS structure that has developmental SA dependence on this neurotransmitter. Agonists at the ionotropic/metabotropic GABA, ACh or Glu receptors can modulate the amplitude or frequency of SA, but blockers at those receptors do not influence SA, suggesting that those transmitter systems are not required for SA (Hunt et al., 2006a). Use of specific receptor antagonists has demonstrated that 5HT_2A/C_ receptors are required for SA; these receptors are localized in the mantle layer of the hindbrain containing newly differentiated neurons and axons (including expression in 5-HT neurons), and are widely distributed laterally (Hunt et al., 2006a; Hood, unpublished data). Although different classes of 5HT neurons co-express other transmitters (glutamate, tachykinins, GABA), there seems to be no role for those transmitters in regulation of hindbrain SA. mRNA for synaptic machinery important to the serotonergic system is expressed at this early developmental stage (e.g., VMAT2, synaptotagmin, VGLUT3; Allen Brain Atlas), suggesting that vesicular release is developing. Thus, 5-HT is expressed and likely is available for release by stage E11.5. Another possibility is the well-characterized volume (extrasynaptic) release of 5-HT; this release may be from the cell body, and may include release from exosomes carrying other cargo such as receptors or adhesion proteins (Borroto-Escuela et al., 2015).

Spontaneous propagating waves in the retina change in transmitter sensitivity as the retina develops (nACh + adenosine early, iGluR + mACh later); however, even with transmitter switching, a combination of ionotropic and metabotropic receptor activation leads to a relatively consistent velocities of waves over the time period of SA (Firth et al., 2005). In developing cortex at P0, waves are initiated by GABAergic circuits, while at P5–6 waves are glutamate initiated. At stages later than P6, the chloride gradient has matured and GABA is inhibitory to waves, as the blocker picrotoxin induces waves. Interestingly, at intermediate stages (P1–P4), both initiating systems co-exist (Easton et al., 2014). Unlike retina and cortex, the receptor profile for SA in the hindbrain does not change over the developmental window of SA, with the exception of a slight contribution of NE receptor signaling at E13.5 (Hunt et al., 2006a).

## Propagation

Events initiating at the InZ propagate into surrounding regions: waves with high calcium intensity move rostrally and caudally along the midline, while waves with lower calcium intensity propagate laterally (Figure 1). At E11.5, waves traveling rostral to caudal from the InZ propagate at 174 ± 16.6 μm/s, while those moving caudal to rostral propagate twice as fast (397 ± 6.45 μm/s; Hunt et al., 2006b); the mechanism behind this difference is not known. These propagation rates are similar to those observed in propagation of retinal waves (150–300 μm/s; Zhou and Zhao, 2000; Firth et al., 2005), but considerably slower that those in the ventral region of the developing cortex (7.2 ± 0.84 mm/s; Conhaim et al., 2010). In embryonic mouse spinal cord waves propagate at rapid rates bidirectionally at E14.5 (Yvert et al., 2004).

At E13.5, the frequency of SA waves is decreased, propagation in the caudal to rostral midline is less frequent than at E11.5, and the propagation speed in each direction is 125 μm/s, demonstrating that the mechanism of rapid rostral-ward propagation has disappeared at this stage (Hunt et al., 2006b). As in the developing retina (Firth et al., 2005), as more cells become refractory to invasion of SA (in the hindbrain *via* gradual spatiotemporal hyperpolarization, see below), both the propagation rate and the frequency of events decrease.

Hindbrain SA is mediated in part by gap junction channels, as non-specific inhibitors of those channels block the waves of activity (Hunt et al., 2006a). The molecular identity of the specific connexin(s) is unknown. Levels of gap junctional coupling are likely important in initiation and conduction of events, as low cell-cell coupling in the midline allows cells to maintain high resistance and enhance spontaneous depolarizations, while increased coupling in lateral tissue may mediate wave propagation into that region (Moruzzi et al., 2009). Gap junctional coupling plays an important role in wave propagation in the developing retina, with early waves driven by gap junctional coupling later supplanted by transmitter-based waves (Zhou and Zhao, 2000; Blankenship et al., 2011).

We postulate that SA event propagation derives from a combination of 5-HT_2A/C_ receptor signaling and gap junctional coupling, with the two mechanisms differing in importance at different positions. In midline cells, *t*-type Ca channels initiate depolarizing events; these cells have relatively high input resistance, and at E11.5 are relatively depolarized. Wave invasion into midline cells may be attributed to gap junctional conduction between the few cells (average cluster size is 8.42 ± 0.74 cells), as even a small junctional current could lead to depolarization and propagation of the event in the similarly high-resistance follower cells. Electrical events in single midline cells have a rapid phase, followed by a sustained plateau of depolarization; we postulate that the rapid phase is mediated by inactivating *t*-type Ca channel currents (Moruzzi et al., 2009), while the slower plateau may be the reflection of currents through gap junctions.

Less intense waves moving into lateral regions propagate at 241 ± 18 μm/s (*n* = 11; Hunt et al., 2005), within the range of wave propagation velocities in the midline of the hindbrain and in the retina. In lateral cells, wave propagation may be mediated by a combination of gap junctional conduction and receptor signaling; lateral cells have lower resistance and do not appear to express *t*-type Ca channels, but have relatively high expression of 5-HT_2A_ receptors (Moruzzi et al., 2009; Watari et al., 2013). Lateral cells are more coupled to their neighbors (average cluster size is 24.3 ± 3.4 cells). Patch clamp recording of lateral events demonstrate monophasic events twice as long as in midline cells. Thus, in lateral tissue, electrical events might transfer between cells *via* gap junctions, and the rise in [Ca]_i_ may be attributed to a combination of intercellular [Ca]_i_ entry through gap junctions and release of intracellular stores via receptor signaling.

## Retraction and Cessation of SA

Waves of activity propagate within the entire hindbrain at E11.5, moving both along the midline and into lateral regions (encompassing trigeminal and facial motor neurons; Gust et al., 2003). By E12.5, waves no longer invade lateral regions, and by E13.5, only the rostral InZ remains active. By E14.5, all waves disappear completely (Figure 1; Hunt et al., 2006b). In order to examine the mechanism of this spatiotemporal retraction, patch clamp recordings were made over the developmental window of retraction, matched to the retracted sites: lateral regions that undergo retraction between E11.5–12.5; midline caudal regions that undergo retraction between E12.5–13.5; and the midline InZ which is silenced at E14.5 (Figure 1).

Recordings within each area demonstrated gradual up-regulation of a K conductance leading to spatiotemporal hyperpolarization of the hindbrain, beginning in lateral regions and ending at the InZ (Figures 1D,E; Watari et al., 2013). SA wave invasion is prevented by this increase in K conductance, which eventually terminates SA *via* decreased resistance and hyperpolarization of the InZ cells to below the voltage range of the window current of *t*-type Ca channels (Watari et al., 2014). Biophysical characterization of the conductance demonstrates voltage- and time-dependence, selectivity to K, and permeation of Cs, similar to currents of the K_2P_ class. We are currently identifying likely candidates mediating the spatiotemporal pattern of hyperpolarization.

The termination of SA in the brainstem, via K-conductance induced hyperpolarization, is unique when compared to other regions where SA has been recorded. In other systems (hippocampus, cortex), SA ends when an excitatory input (depolarizing GABA) becomes inhibitory as the adult form of the Cl- gradient is established, or when transmitter systems mature. In mouse spinal cord, the interval between spontaneous episodes increases between E11.5–14.5 (Hanson and Landmesser, 2003), becoming infrequent at E15.5, in parallel with the Cl^−^ gradient (Delpy et al., 2008). At the same time episodes show localization, becoming more frequent in cervical regions (Yvert et al., 2004).

## Midbrain SA and Looping

SA waves initiated at the InZ propagate caudally before ceasing near the rostral end of the spinal cord. At the rostral end of the hindbrain, waves approach the isthmus, the hindbrain/midbrain border. A substantial proportion of hindbrain-initiated waves propagate across the isthmus into the ventral tegmentum, and from there, dorsolaterally into the tectum (Rockhill et al., 2009). The waves eventually extinguish in the dorsolateral area. The ability to cross is stage-dependent, as events in E12.5 embryos propagate across the isthmus more frequently, and further into the midbrain; at E13.5, fewer events occur in the hindbrain, and a lower proportion of those invade the midbrain. Waves move dorsolaterally in the midbrain at a speed of 184 μm/s.

In 80% of the E12.5 litters, waves in half of the embryos demonstrate a looping pattern (Figure 2); in these InZ-initiated events, waves propagate faster along one side of the midline, which has two distinct tracks. At the isthmus, the leading side of the event crosses into the midbrain and exits out the isthmus into the other track. The event can subsequently re-activate the isthmic region, causing repetitive loops of excitation that move either across the midbrain tegmentum (loops of 5–6 s duration), or cross the midline within the most rostral region of the hindbrain (loops of 3 s duration; Figure 2A). These repeated loops of activity can last for more than 20–30 min. The defining characteristic of these loops is the onset of a new event before the previous event dissipates *via* [Ca]_i_ extrusion or re-uptake, effectively leaving [Ca]_i_ at relatively high levels for the duration of the loop (Watari et al., 2014). At the isthmic midline, *t*-type Ca channels are transiently up-regulated at E12.5, making this region excitable, and allowing both the crossing of individual events and the ability of the loops to form. In midbrain loops, the wave travels over regions where dopamine neurons are differentiating, and the burst of [Ca]_i_ may play crucial roles in their differentiation; it may also direct the rostrally-directed axons of 5-HT neurons (Figure 2B). In rostral hindbrain loops, the wave passes over the area containing newly differentiated 5HT neurons. The repeated oscillations in [Ca]_i_ may influence the differentiation or axon extension of these neurons. These loops are expressed exclusively at E12.5, as expression of *t*-type Ca channels is up-regulated at that point. By E13.5, the two-sided track of the hindbrain midline has coalesced into a single pathway, and events do not have a return pathway, terminating the looping pattern (Watari et al., 2014).

## Discussion

Propagating waves of SA are independent of sensory input or higher commands, and in many brain states, would not be compatible with adult information processing. Hindbrain and midbrain cells that previously participated in waves of SA alter their embryonic forms of excitability as they become mature. Interestingly, the cells do not appear to simply downregulate the original conductance that allowed participation in the waves, as K-induced depolarization of the newly quiescent hindbrain at E15.5 allows spontaneous events that propagate in the midline in a manner similar to that seen during the period of SA. Thus, up-regulation of a resting conductance is an elegant way to for cells to transition from embryonic to mature physiological activity, while allowing them to use channels involved in excitation when appropriate to do so.

The expression of SA in the brainstem during differentiation and axon extension of different classes and types of neurons suggests that propagating waves may influence maturation steps of these cells. We are using cultured explants of tissue to determine what role SA plays in specification, differentiation or axonal pathfinding of neurons that experience these waves.

## Ethics Statement

Experiments reported here were carried out on a protocol that was reviewed and approved by the University of Washington IACUC, according to their guidelines.

## Author Contributions

MMB designed the research, performed experiments, performed analysis and interpretation of the data and prepared the manuscript.

## Funding

Supported by: NSF grant #IOS-095295; NIH grant #MH104773-02.

## Conflict of Interest Statement

The author declares that the research was conducted in the absence of any commercial or financial relationships that could be construed as a potential conflict of interest.
